# Study on Key Properties and Model Establishment of Innovative Recycled Aggregate Pervious Concrete

**DOI:** 10.3390/ma17143535

**Published:** 2024-07-17

**Authors:** Panfeng Zhao, Jingfei Zhou, Zhengnan Zhang, Shoukai Chen

**Affiliations:** 1 Sinohydro Foundation Engineering Co., Ltd., Tianjin 301700, China; lyon9709@163.com; 2 School of Water Conservancy, North China University of Water Resources and Electric Power, Zhengzhou 450046, China; z202210010090@stu.ncwu.edu.cn; 3 Henan Key Laboratory of Water Environment Simulation and Treatment, Zhengzhou 450045, China; 4 Nanjing Hydraulic Research Institute, Nanjing 210024, China; zhengnan_zhang@163.com

**Keywords:** innovative recycled aggregate pervious concrete, response surface methodology, compressive strength, permeability coefficient, acrylic pipe

## Abstract

In order to meet the needs of low-impact development and sustainable development, there is an urgent desire to develop an innovative recycled aggregate pervious concrete (I-RAPC) that is of high strength and permeability. In this study, I-RAPC was prepared based on response surface methodology (RSM) using recycled aggregate, river sand, and different types of pipes as the materials, and the effects of different pipe parameters (number, diameter, material, and distribution form) on the performance of I-RAPC were investigated. In addition, the calculation model of the compressive strength and the permeability coefficient of I-RAPC were proposed. The results showed that the frontal- and lateral-compressive strengths of I-RAPC were 39.8 MPa and 42.5 MPa, respectively, when the pipe material was acrylic, the position was 1EM, and the diameter was 10 mm—at which time the permeability coefficient was 3.02 mm/s, which was the highest in this study. The maximum relative errors of the compressive strength calculation model and the permeability coefficient calculation model were only 7.52% and 4.42%, respectively, as shown by the post hoc test. Therefore, I-RAPC has the advantages of high strength and permeability and is expected to be applied in low-impact development in cities with heavy surface sediment content and rainfall.

## 1. Introduction

Since the start of the 21st century, human beings have been faced with problems of a lack of natural resources and environmental deterioration, such as urban flooding, water pollution, and other phenomena that occur frequently all over the world [1]. At the same time, with the rapid development of urban construction, there has also been a large production of construction waste. According to statistics, the United States, China, and India are the three main producers of construction waste, and their total annual output has been close to 8 billion tons [2]. The traditional method of construction waste disposal generally chooses landfilling or stacking, which wastes limited land resources and has a great impact on the environment [3]. What is more, high-end waste monitoring technologies, such as geographic information systems (GIS), radio-frequency identification (RFID), ultrasonic sensors, and international mobile/general radio packet service systems (GSM/GPRS), are mostly not suitable for poor countries due to their high costs [4]. If construction waste is simply discarded as waste, it will result in a deplorable waste of resources. Comprehensively speaking, the resource utilization of construction waste is one of the most realistic ways to achieve the world’s strategic pursuit of environmental protection and sustainable development.

Whenever urban flooding in recent years is mentioned, it is believed that these problems are caused by the untimely infiltration of rainwater. It can be seen that excellent permeability is crucial for pervious concrete. Unlike ordinary concrete that needs to be impermeable, pervious concrete needs pores to ensure that enough water can seep down in a short period of time [5,6]. Pervious concrete has an interconnected pore structure, which can make rainfall infiltrate rapidly. The compressive strength and the permeability coefficient of pervious concrete are 5.5~40 MPa and 0.3~14 mm/s, respectively. Because of its good permeability, it has been applied to the construction of low-impact development [7,8]. The combination of construction waste and pervious concrete to prepare recycled aggregate pervious concrete (RAPC) can not only effectively alleviate urban flooding and the urban heat island phenomenon but also reduce the adverse impact of construction waste on the environment. After the continuous research of many scholars in recent years, the related technologies of recycled aggregate and recycled aggregate pervious concrete are becoming more and more perfect but the problems existing in the practical application of RAPC are also gradually emerging. First, there is a negative correlation between the strength and porosity of RAPC, which makes it difficult to have high strength and permeability at the same time, limiting the application range of RAPC [9]. On the other hand, typical pervious concrete pavement has some drawbacks, including pore blockage, limited durability, and complex maintenance [10,11]. Zhu et al. [12] added polypropylene thick fibers (PPTF) and copper-coated steel fibers (CCF) to pervious concrete and found that permeability was affected by fiber diameter and that the coefficient of permeability was related to tortuosity and porosity. Park et al. [13] used coal bottom ash aggregate as raw material to control the permeability of pervious concrete by adjusting the water/cement (W/C) ratio and compaction level and found that the compaction level was negatively correlated with the permeability coefficient. El-Hassan et al. [14] designed different recycled aggregate substitution rates to investigate the effect of recycled aggregate substitution of natural aggregate on pervious concrete, and the results showed that an increase in the recycled aggregate substitution rate led to an increase in the permeability coefficient of RAPC. Numerous studies have shown that the permeability of traditional pervious concrete is affected by many aspects, and it is very hard to stabilize and control its permeability. Therefore, based on traditional permeable concrete, it is particularly important to explore a new type of pervious concrete with high strength and high permeability at the same time to solve the problems existing in RAPC [15,16,17].

Up until the present moment, there has been little research on this new type of pervious concrete. For example, Niu et al. [18] prepared pervious concrete by artificially creating regular pipes connected penetrably in the concrete instead of the previous uniformly distributed pore structure. It is applied to different heavy rainfall levels, and the relationship between porosity and heavy rainfall level is established with the help of the water permeability coefficient, which provides a basis for the surface design of a new type of pervious concrete pavement. Zhu et al. [19] used the same method to make a new high-strength straight-hole recycled pervious concrete (HSRPC) for secondary highway pavement and used surface water depth and drainage time to describe the flooding resistance of HSRPC. Li et al. [20] manufactured high-strength pervious concrete (HSPC) pavement, which showed a compressive strength as high as 61.37 MPa within 7 days and a corresponding permeability coefficient of 13.02 mm/s. A drainage system was designed to remove the blocked dust in the pores of HSPC pavement. Therefore, compared with traditional pervious concrete, this innovative high-strength pervious concrete has more extensive application potential. However, their current research is still focused on the relationship between porosity, strength, and water permeability of the concrete. The strength and permeability of this type of pervious concrete are determined by the pore characteristic parameters (e.g., quantity, diameter, material, and location) of the pipeline, and there is also a certain relationship between each factor. How to quantify the correlation between the characteristic parameters of the new pervious concrete pipe and its performance to provide prediction guidance for practical engineering applications is a missing part of the current research. Therefore, in this paper, tubular products of different materials are embedded in the upper and lower connected pipes to prepare innovative pervious recycled aggregate concrete (I-RAPC), to analyze the influence of several characteristic parameters on the performance of I-RAPC.

Response surface methodology (RSM) can directly obtain the influence of a multi-factor interaction on the performance of concrete through the response surface model and effectively predict the response results, which has the advantages of fewer test times, a short period, high precision, and a wide application range [21,22,23,24]. RSM tests the representative local test sites and obtains the data, and the functional relationship between the factors and the result in the whole range is obtained by regression fitting [25]. Finally, the optimal level value of each factor for the response result can be obtained through optimization [26,27,28].

The above research and application show that I-RAPC has the advantages of high strength and high permeability compared with RAPC. However, at present, there is not enough research to explain the influence of different pipeline characteristic parameters on the performance of I-RAPC. Therefore, this paper focuses on the relationship between I-RAPC strength, permeability, and pipe characteristics and analyzes the relationship between each influencing factor and performance by the RSM to construct the prediction model of the I-RAPC performance index. At the same time, the theoretical calculation formula of the I-RAPC permeability coefficient is deduced, and the formula is verified by experiments. The research results can provide a theoretical basis for the popularization and application of I-RAPC and help to promote the resource utilization of construction solid waste.

## 2. Materials and Methods

### 2.1. Main Raw Materials and Mix Design

The recycled coarse aggregates (RCA) from a C25 waste concrete pavement were obtained by crushing and sieving waste concrete. Considering that the prepared I-RAPC pipes had a diameter of 5–10 mm, RCAs with particle sizes of 4.75–9.5 mm were chosen to decrease the negative impact (such as roughness and compactness) of large particle size aggregates on the concrete sidewall of the pipe. Table 1 illustrates the indexes of RCA, which meet the criteria of the Chinese national standard JGJ/T 240-2011 [29]. In particular, the numerical tube pressure was determined in accordance with the Chinese national standard GB/T 17431.2-2010 [30]. The fine aggregates were continuous-grade natural river sand with a fineness modulus of Mx = 2.9 and an apparent density of 2549.89 kg/m^3^, which is classified as intermediate sand. P·O 42.5 ordinary Portland cement in this study was produced by Tianrui Co., Ltd. (Henan, China), with a specific surface area of 348.7 m^2^/ kg and density of 3120 kg/m^3^. Tap water conforming to the Chinese national standard JGJ 63-2016 [31] was used for mixing, which had a pH of 7.2, a chloride ion content of 0.354 mg/L, and a TDS of 137 ppm. The pipes included acrylic pipes (produced by Weidu Glass Products Co., Ltd. in Henan, China), bamboo pipes (produced by Zhuyuan Bamboo Products Co., Ltd. in Anji, China), and concrete pipes. The pipes that were not embedded in any pipes were defined as the concrete pipe. The modulus of elasticity of acrylic, bamboo, and concrete pipes were 2.33 GPa, 1 GPa, and 3 GPa, respectively [32]. Given that pipe diameter would impact I-RAPC performance and construction ease, the pipe diameters chosen in this study were 5 mm, 7.5 mm, and 10 mm.

According to the Chinese national standard DL/T 5330-2015 [33], the water–cement ratio (W/C) of I-RAPC was 0.49 and the sand ratio was 0.345. The mix design is illustrated in Table 2.

### 2.2. Experimental Design

**Figure 1:**
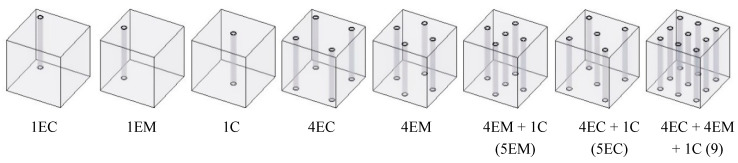
Pipe distribution pattern of I-RAPC.

In this study, the size of the I-RAPC specimen was 150 mm × 150 mm × 150 mm. The reference group did not set pipes, while other concrete specimens were set with 1, 4, 5, and 9 pipes, respectively. The distribution forms include the center (C), middle edge (EM), and corner of edge (EC) distribution forms. The specific distribution forms of pipes are shown in Figure 1. In addition, pipes could lead to differences in compressive strength between different compression surfaces. During the test, the frontal- and lateral-compressive strengths were measured separately, where the frontal- and lateral-compressive strengths refer to the strengths measured perpendicular and parallel to the axis of the pipe.

#### 2.2.1. Design Plan of I-RAPC Response Surface Test

A three-factor, three-level response surface test for I-RAPC was designed using the number, size (diameter), and material (modulus of elasticity, roughness) of the pipes as response factors, and the frontal- and lateral-compressive strengths and permeability coefficient as response values. The I-RAPC style for the number and distribution of pipes numbered 1C, 5EM, and 9 in Figure 1 were selected. Response surface design analysis of I-RAPC was carried out using *Box-Behnken Design* (BBD) in *Design Expert 10.0.3*. The design of the I-RAPC response surface test is shown in Table 3.

#### 2.2.2. I-RAPC Theoretical Model Construction Test Scheme

The I-RAPC compressive theoretical model construction test scheme was determined based on the I-RAPC strength of single pipes of different materials (concrete, acrylic), distinct diameters (5 mm, 10 mm), and different distribution forms (C, EM, MC, etc.). See Table 4 for the specific test plan.

### 2.3. Test Methods

#### 2.3.1. Preparation Process

**Figure 2:**
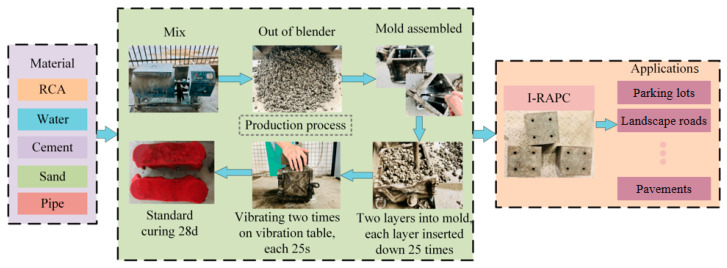
The preparation process of I-RAPC.

The test used the pre-wet aggregate method to mix concrete [34]. Based on the above mix ratio, the recycled coarse aggregate was first mixed with 1/2 water by a mixer to prevent the recycled coarse aggregate from absorbing mixed water in the subsequent mixing and hydration hardening process; then, cement and sand were added, stirred for 1 min, and the remaining water was poured into the remaining water to be mixed evenly and then loaded into a special mold test. Wet towels were placed as covers after manual insertion and mechanical vibration. After indoor maintenance for 24 h, the mold was removed and placed in the standard maintenance room until the specified age. The specific process is shown in Figure 2.

As shown in Figure 2, the pipes in I-RAPC were made by a special mold, and the internal diameters of the selected bamboo pipes and acrylic pipes were the same as those of the iron column arranged in the mold, ensuring that the internal diameters of the pipes in I-RAPC were consistent for each material.

The test indexes included I-RAPC strength performance (frontal and lateral compressive strength) and permeability performance (water permeability coefficient). The specimen was a 150 × 150 × 150 mm standard cube. The average of the measured values of three specimens was used as the test results for the compressive strength and permeability coefficient of each group of specimens. When the difference between one measured value and the intermediate value exceeded 15% of the intermediate value, the intermediate value was taken as the experimental result. When the difference between two measured values and the median exceeded 15% of the median, the experimental data were invalid and needed to be retested to ensure the accuracy of the experimental data.

#### 2.3.2. Compressive Strength Test

Because of the pipe, the I-RAPC compressive area is smaller when the force is parallel to the pipe than it is when it is perpendicular. Consequently, frontal compressive strength and lateral compressive strength are the terms used to characterize the compressive strength tests in this study that have the force directions perpendicular and parallel to the pipe, respectively. The compressive strength test was completed on the DY-3008DX electro-hydraulic servo microcomputer-controlled pressure tester. It should be noted that in order to ensure that the surfaces of the specimens were flat, a smoothing machine was employed to treat the specimens before the frontal compressive strength test. According to the Chinese national standard GB/T 50081-2002 [35], the loading speed was set at 0.5 MPa/s. After the test, I-RAPC compressive strength was calculated according to Equation (1):

(1) $$f_{cu}=\frac{F_{max}}{A}$$

where $f_{cu}$ is the cube compressive strength of the test block, MPa; $F_{max}$ is the maximum load, N; A is the stress area of the I-RAPC, mm^2^, takes 22,500 mm^2^; and the effect of pipe on the bearing surface area is not considered.

#### 2.3.3. Permeability Test

**Figure 3:**
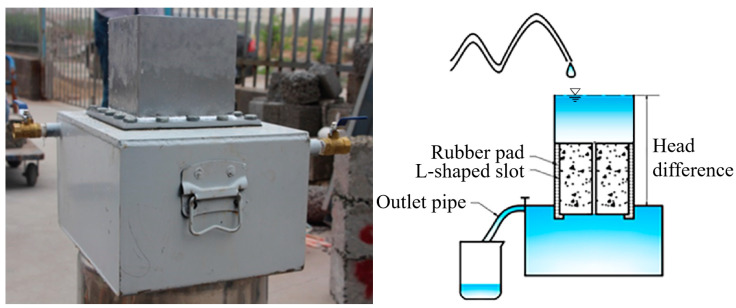
Measuring device for water permeability coefficient.

The permeability coefficient was an intuitive indicator to represent the permeability performance of I-RAPC. This test was based on Darcy’s law constant head method [36] and adopted a self-made permeable device (as shown in Figure 3) in which the head difference *H* was 200 mm. To prevent the leakage of the non-test surface of the test piece from affecting the test data, the gap between the test piece and the permeable device was filled with plasticine. Before the test, the water flow rate was adjusted to make the water stably overflow from the top surface of the device and it was kept stable for 60 s. The test time of each test block was controlled within 25 s. The permeability coefficient of I-RAPC can be calculated according to Equation (2):

(2) $$k=\frac{VL}{AHt}$$

where k is permeability coefficient, mm/s; t is the measurement time, s; V is the volume of water in the beaker at time t, mm^3^; L is the height of the specimen, takes 150 mm; H is the head difference, mm.

#### 2.3.4. Roughness Coefficient Test

The coefficient of roughness is a dimensionless comprehensive coefficient that measures the influence of boundary shape and roughness on water flow resistance. The magnitude of the pipeline coefficient of roughness is mainly related to the surface roughness of the pipe material. Therefore, the engineering community generally infers the coefficient of roughness through a large amount of measured data. In this experiment, the coefficient of roughness values of different pipes were obtained through repeated experiments using the permeable device in Figure 3 and the Chézy–Manning formula, which is as follows:

(3) $$n=\frac{R^{\frac{2}{3}}J^{\frac{1}{2}}}{V_{p}}$$

where n is the roughness coefficient; R is the hydraulic radius, m; J is the hydraulic slope; $V_{p}$ is the average flow velocity of the pipeline cross-section, m/s.

## 3. Results and Discussion

### 3.1. Response Surface Methodology (RSM) Analysis

#### 3.1.1. Response Surface Model Construction and Analysis

According to the Weierstress polynomial optimal approximation theorem, most functions can be approximated by polynomials, and the polynomial approximation model can deal with a wide range of nonlinear problems. Therefore, in practical application, regardless of the relationships between independent variables and dependent variables, the polynomial approximation model can be used for analysis [37,38,39].

Multiple regression fitting analysis was carried out for the frontal- and lateral-compressive strengths and the permeability coefficient, respectively, and multiple regression equations of actual values were obtained as shown in Equations (4)–(6):

(4) $$f_{cu-front}=61.30565-3.42578x-1.98603d-11.8321E+0.4375xE+0.70417dE+0.1283x^{2}+1.46802E^{2}$$

(5) $$f_{cu-side}=44.31826-1.78045x+0.09d-10.12039E-0.102xd+2.63789xE-0.79244xE^{2}+3.25463E^{2}$$

(6) $$k_{actural}=-39.36292-1.86225x+10.99522d+3951.26057n+0.4346xd+184.89167xn-\;1195.04989dn-0.69725d^{2}+32227.75322n^{2}-14356.66667xn^{2}+75.94873d^{2}n$$

where $f_{cu-front}$ is the actual value of I-RAPC frontal compressive strength, MPa; $f_{cu-side}$ is the actual value of I-RAPC lateral compressive strength, MPa; $k_{actural}$ is the actual value of the I-RAPC permeability coefficient, mm/s; x is the number of pipes; E is the elastic modulus of the pipe, GPa; n is the roughness coefficient of the pipe.

Analysis of variance and significance tests were conducted for the regression equations above, as listed in Table 5. The values of *p* in the model are all less than 0.05, so the model is significant and can be used in subsequent optimization design. The lack-of-fit items indicate the degree of fit between the model and the test, i.e., the degree of difference between them [40]. The values of *p* of the lack-of-fit items in the model were all greater than 0.05, indicating that there was no significant discrepancy between the model and the test, that is, the degree of non-correlation between the test data and the model was not obvious, and the model was credible.

**Figure 4:**
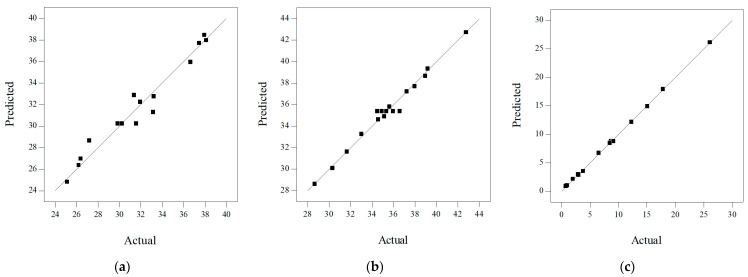
Distribution diagram of model’s actual values and predicted values: (**a**) frontal compressive strength; (**b**) lateral compressive strength; (**c**) permeability coefficient.

Table 6 shows the statistical analysis results of the regression equation error of I-RAPC frontal- and lateral-compressive strength and the permeability coefficient. The model determination coefficients *R*^2^ were 0.9524, 0.9573, and 0.9993 respectively, indicating that the predicted values of the model were in good agreement with the actual values. The model correction determination coefficients *Adj R*^2^ were 0.9153, 0.9240, and 0.9983, i.e., the model regression equation could simulate 91.53%, 92.40%, and 99.83% of the changes in response values. Additionally, the absolute values of the difference between the model correction determination coefficient *Adj R*^2^ and the model prediction determination coefficient *Pred R*^2^ were 0.1388, 0.1795, and 0.0408, which were less than 0.2, proving that the regression model could fully explain the process problems [41]. It is generally considered reasonable to have *Adeq Precision* greater than 4 [42]. In this paper, *Adeq Precision* were 16.57, 22.923, and 113.46, respectively. In addition, the coefficient of variation *C.V.* of the model was 3.79%, 2.55%, and 3.27%, less than 10%, which indicated that the test had high accuracy and reliability [43]. Therefore, the regression equation of the model could replace the actual value of the test and analyze the test results. Figure 4 indicates the distribution of actual and predicted values of the response model. The corresponding point between the actual and predicted values was near the line *y = x*, i.e., the predicted values of the compressive strength response model were close to the actual values and had good agreement.

The experimental results and RSM model showed that the I-RAPC with the bamboo pipe was lower than the I-RAPC with the other two pipes (concrete, acrylic). The reason for this might be that bamboo pipes had a low elastic modulus and high coefficient of roughness but the compressive strength of I-RAPC was proportional to elastic modulus within a certain range, and the permeability coefficient was inversely proportional to the pipe coefficient of roughness. Therefore, only the I-RAPC performance test results of concrete pipes and acrylic pipes were compared and analyzed with the optimization prediction results of the response surface model.

#### 3.1.2. Response Surface and Contour Map

**Figure 5:**
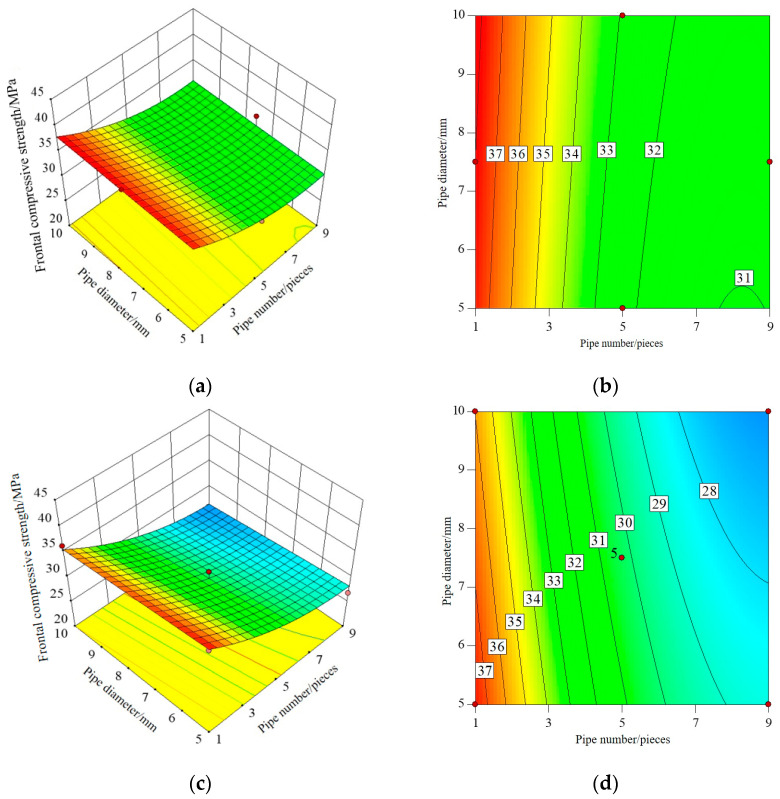
The interaction effect of the number and diameter of the pipes on the frontal compressive strength of I-RAPC: (**a**) the response surface (E = 3 GPa); (**b**) the contour (E = 3 GPa); (**c**) the response surface (E = 2.33 GPa); (**d**) the contour (E = 2.33 GPa).

**Figure 6:**
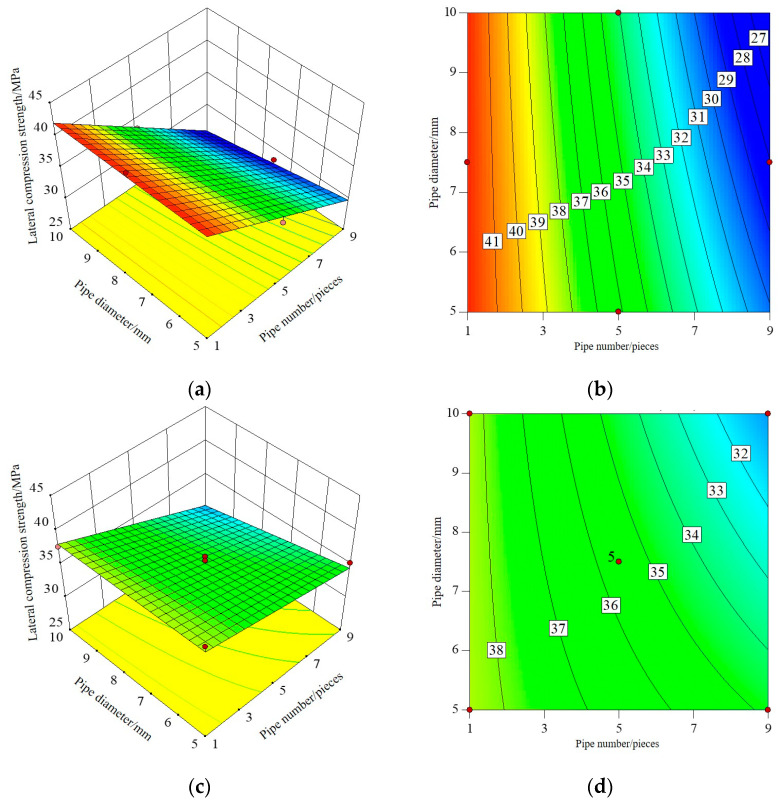
The interaction effect of the number and diameter of pipes on the lateral compressive strength of I-RAPC: (**a**) the response surface (E = 3 GPa); (**b**) the contour (E = 3 GPa); (**c**) the response surface (E = 2.33 GPa); (**d**) the contour (E = 2.33 GPa).

**Figure 7:**
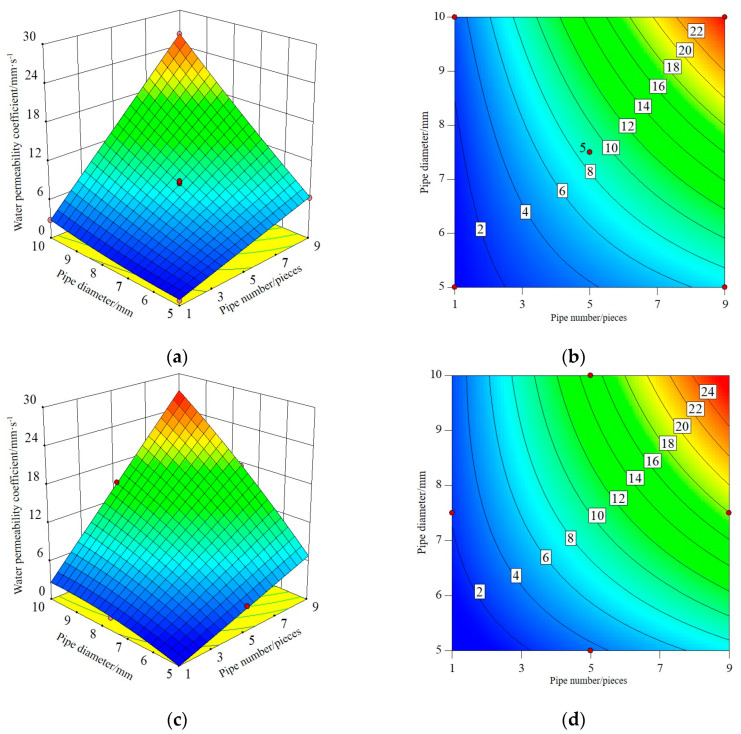
The interaction effect of the number and diameter of the pipes on the water permeability coefficient of I-RAPC: (**a**) the response surface (*n* = 0.01); (**b**) the contour (*n* = 0.01); (**c**) the response surface (*n* = 0.0075); (**d**) the contour (*n* = 0.0075).

The three-dimensional response surface and contour map established by RSM can directly reflect the interaction between test factors, that is, when one factor is a certain value, the interaction between the other two factors affects the response value. The shape of the contour line can reflect the strength of the interaction effect. An ellipse indicates that the interaction between the two factors is significant, while a circle is the opposite. Among the three influence factors, the number and diameter of pipes have the greatest influence on I-RAPC strength and the permeability coefficient. Therefore, the interaction of the number and diameter of pipes on I-RAPC strength and the permeability coefficient will be discussed in this part, and its response surface and contour diagram are illustrated in Figure 5, Figure 6 and Figure 7.

Figure 5 shows the interactive effect of the number and diameter of pipes on the front compressive strength of I-RAPC. From the response surface, the frontal compressive strength of I-RAPC decreases with the increase in the number and diameter of pipes, and the effect of the number of pipes on the frontal compressive strength is greater than the diameter of pipes. At the same time, by observing the mapping distribution of the response surface, it is found that the contour curvature in this range is relatively small and the degree of bending is low. Therefore, the interaction between the number and diameter of pipes on the frontal compressive strength of I-RAPC is small [44]. At the same time, it is found that the frontal compressive strength of the I-RAPC without the acrylic pipe is slightly higher than that of the I-RAPC when the acrylic pipe is embedded.

Figure 6 shows the interactive effect of the number and diameter of pipes on the lateral compressive strength of I-RAPC. From the response surface, the lateral compressive strength of I-RAPC decreases with the increase in the number and diameter of pipes, which is similar to that of the front compressive strength. At the same time, by observing the distribution of the response surface on the coordinate axis, it is found that the contour lines obviously bend when the acrylic pipe is embedded, and the influence of the number and diameter of pipes on the lateral compressive strength of I-RAPC is interactive. At the same time, compared with the side compressive strength response surface of I-RAPC without embedded pipe, it is found that when the number of pipes is large, the embedded acrylic pipe can slightly improve the lateral compressive strength of I-RAPC.

Figure 7 shows the interactive effect of the number and diameter of pipes on the permeability coefficient of I-RAPC. Through the distribution of the response surface, it can be seen that both the number and diameter of pipes have a great influence on the permeability coefficient, and the contour curvature is larger, indicating that the interaction of the two factors on I-RAPC is significant, and the higher the permeability coefficient is with the increase in the number and diameter of pipes. At the same time, it is found that the embedded acrylic pipe can significantly increase the permeability coefficient of I-RAPC. When the number of pipes reaches nine and the diameter of pipes reaches 10 mm, the permeability coefficient can reach 27.144 mm/s.

#### 3.1.3. The Optimized Prediction Results Compared with the Actual Results

The optimization option in *Design-Expert 10.0.3* software was utilized to set the input conditions (number of pipes, diameter, elastic modulus of material/coefficient of roughness) for solving, and the target response result and its suitability were obtained. With the highest response value as the ultimate goal, the output range of compressive strength was set to be 0~45 MPa and the output range of the permeability coefficient was set to be 0~30 mm/s. The conditions for outputting the results were as follows:

(1) Frontal compressive strength: the number of pipes was one, the diameter of pipes was 5 mm, and the modulus of elasticity of the pipes was 3 GPa, its absolute error was 0.61 MPa, and the relative error was 1.46%;
(2) Lateral compressive strength: the number of pipes was one, the diameter of the pipes was 5 mm, and the modulus of elasticity of the pipes was 3 GPa, its absolute error was 1.97 MPa, and the relative error was 4.49%;
(3) Permeability coefficient: the number of pipes was nine, the diameter of the pipes was 10 mm, and the roughness of the pipes was 0.0075, its absolute error was 0.09 MPa, and the relative error was 0.33%.

It could be seen that the suitability of the optimized models are all excellent. The absolute error ranges between the optimized prediction results and the actual results are 0.03~6.61 MPa and 0.004~0.310 mm/s, and the corresponding relative error ranges are 0.10~27.30% and 0.33~9.48%. The experimental results are similar to the model optimization prediction results, and the relative errors of most indexes are within 15%, which indicates that the design experiment based on the response surface analysis method has practical significance and holds the advantage of less data required; however, there are a few cases where the predicted values of the model differ greatly from the actual values. This is due to the response surface design, which reduces the amount of experimentation that results in a loss of accuracy. Therefore, the response surface method can first implement a simple test design and law summary for the uncertain tests and, then, further implement fine tests to explore its essential law, which has a certain guiding significance for test and law exploration.

### 3.2. Compression Strength and Failure Morphology

#### 3.2.1. The Optimized Prediction Results Compared with the Actual Results

**Figure 8:**
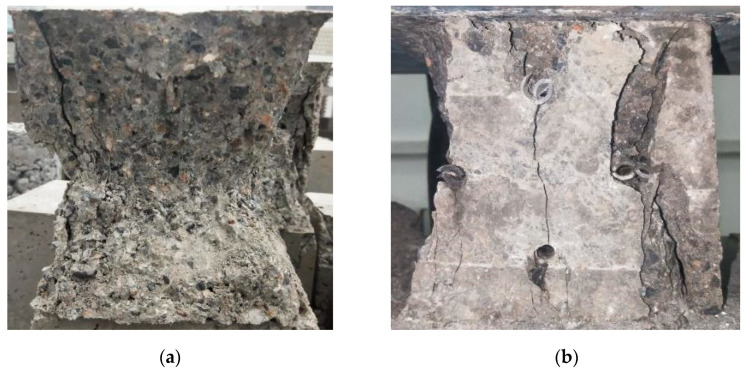
The failure of morphology of I-RAPC: (**a**) frontal compressive strength; (**b**) lateral compressive strength.

During the frontal compressive strength test, cracks gradually appeared on the surface of I-RAPC with the increase in load, then the cracks started from the middle of the specimen and expanded to the outer wall of the pipe and the edge of the specimen, finally forming the “8” shaped failure morphology as shown in Figure 8a. During the lateral compressive strength test, cracks first appeared on the surface of the concrete and kept peeling off, then perpendicular and penetrating cracks appeared between the pipes, followed by deformation of the pipes, and finally the specimens lost their compressive capacity at the maximum load and the specimens were damaged after unloading (as shown in Figure 8b). It could be seen that the presence of pipes changed the internal structure and failure morphology of concrete, and the failure morphology of I-RAPC was different from that of other pervious concrete. Compared with other pervious concrete, I-RAPC was less likely to be damaged due to the fact that in the profile, the shape of the pipe was circular and had a better pressure-bearing effect. According to the Chinese national standard DL/T 5330-2015 [33], the compressive strength of standard concrete was approximately 36.1 MPa. With this result as the forward compressive strength target, the arrangement should be selected as 1EM, 1EC, and 1C; accordingly, the specimen could satisfy the demand of lateral compressive strength greater than 36.1 MPa when the arrangement was 1EM-T, 1EC, 1EM-S, and 1C.

**Figure 9:**
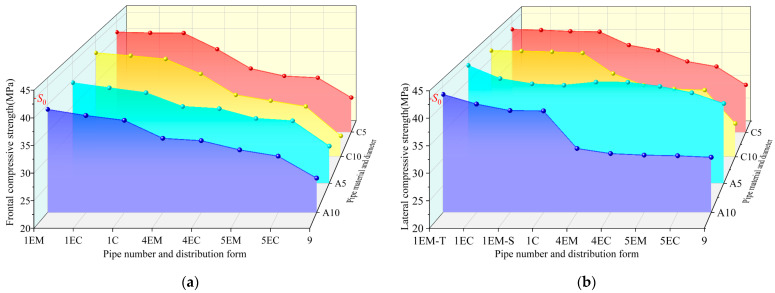
Effects of pipes on compressive strength of I-RAPC: (**a**) frontal compressive strength; (**b**) lateral compressive strength.

The I-RAPC compressive strength test results are illustrated in Figure 9, where C5, C10, A5, and A10 represent 5 mm and 10 mm concrete and acrylic pipe pores, respectively. The figure illustrates that I-RAPC compressive strength decreases as the number of pipe pores and the diameter of the pipe pores increase, which is consistent with the trend that the strength of ordinary recycled pervious concrete decreases as porosity increases. The main reason for the analysis is that the existence of the form of pipes destroys the pressure-bearing structure of the original dense concrete and forms a weak surface inside the concrete, so more pipes are disadvantageous to the bearing pressure of concrete. Even so, the measured compressive strength is still much higher than that of RAPC [15]. The position of the pipes will affect the frontal compressive strength 1EM > 1EC > 1C and the lateral compressive strength 1EM-T > 1EC > 1EM-S > 1C for I-RAPC with the same number of pipes. In the case of multi-pipes, there are also 4EM > 4EC and 5EM > 5EC characteristics. That is to say, the lower compressive strengths of I-RAPC occur when the position of the pipe is closer to the center of the concrete relative to the bearing surface. When a single pipe (such as the position of the EM-T) was filled with acrylic pipe (5 mm in diameter), the I-RAPC lateral compressive strength of the concrete pipe increased by 1.0 MPa. Acrylic pipes can significantly enhance I-RAPC, and the maximum compressive strength can be increased by 5.5 MPa.

**Figure 10:**
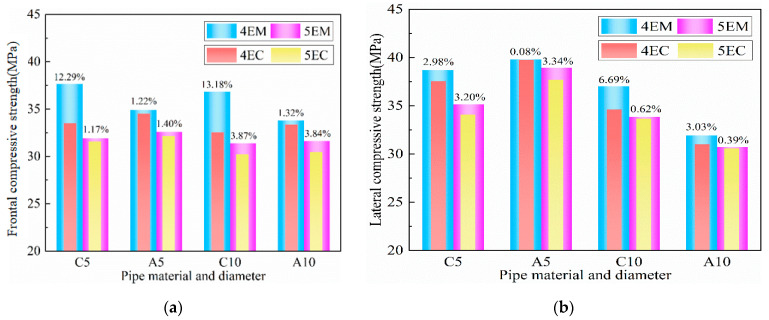
Degrees of improvement from EM pipe distribution on I-RAPC compressive strength: (**a**) frontal compressive strength; (**b**) lateral compressive strength.

In addition, different pipe locations with the same number of pipes also have certain effects on I-RAPC. For example, the distribution of EM pipes is better than that of EC, and its improvement of the compressive strength of I-RAPC is shown in Figure 10. Compared with EC, EM could increase the compressive strength by about 0.08~13.18%. Therefore, it is recommended to design I-RAPC with EM when higher strength is the target.

There is a basic inverse relationship between porosity and strength of most solid materials [40,41], as shown in Equation (7):

(7) $$S=S_{0}e^{-kp}$$

where S is the compressive strength of the material when porosity is *p*, MPa; $S_{0}$ is the intrinsic strength when porosity is zero, MPa; k is a constant; *p* is the porosity, %.

**Figure 11:**
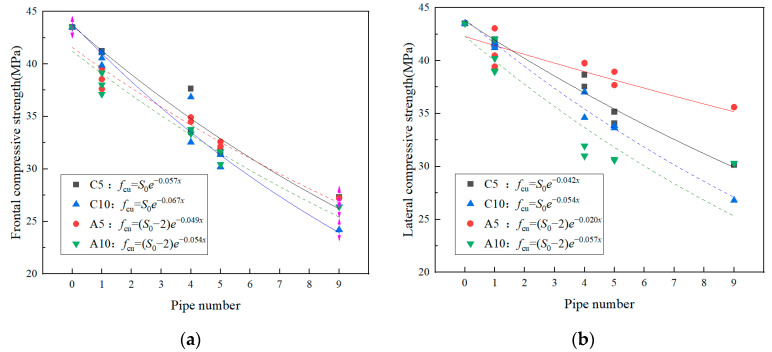
Fitting results of I-RAPC compressive strength and pipe number: (**a**) frontal compressive strength; (**b**) lateral compressive strength.

The fitting results of I-RAPC compressive strength and number of pipes were obtained by the orthogonal distance regression method, as illustrated in Figure 11.

According to Equation (7), the general expression of the relationship between the I-RAPC strength of different pipe diameters and materials and the number of pipes is shown in Equations (8) and (9).

Concrete pipe I-RAPC:

(8) $$S=S_{0}e^{-kx}$$

Acrylic pipe I-RAPC:

(9) $$S=\left(S_{0}-2\right)e^{-kx}$$

where S is the compressive strength of the material when the number of pipes is *x*, MPa; $S_{0}$ is the intrinsic strength when the number of pipes is zero, MPa, which was 43.5 MPa in this study; k is a constant, which is related to the pipe material, diameter, and I-RAPC strength type, specific k values are shown in Figure 11.

#### 3.2.2. Theoretical Analysis

According to the test results, the more pipes the I-RAPC has, the larger the pore diameter, and the lower the compressive strength. Therefore, the strength reduction coefficient method is proposed, and the theoretical calculation model of I-RAPC compressive strength and pipe parameters is constructed.

The corresponding strength reduction factor *c* was defined as the ratio of the I-RAPC strength of concrete single pipes at different positions to the strength of the reference group, as shown in Equation (10). The strength characteristics of I-RAPC were changed when the acrylic pipe was inserted into the pipe, so the reduction degree was calculated using the corresponding strength of the concrete pipe, as shown in Equation (11). The strength reduction coefficients of concrete pipes at various positions are calculated using Equation (10), and the results of the calculations are shown in Table 7.

(10) $$c=P_{c}/P$$

(11) $$a=P_{a}/P_{c}$$

where c is the strength reduction factor of the concrete single pipe; $P_{c}$ is the I-RAPC strength of concrete single pipes in different positions, MPa; *P* is the average strength of the reference group, MPa, which is 43.5 MPa in this study; a is the strength reduction factor of the acrylic single pipe; $P_{a}$ is the I-RAPC strength of acrylic pipe in different positions, MPa.

The theoretical calculation model of I-RAPC compressive strength with different pipe parameters was established as follows:

(12) $$f_{{cu}_{c}}=P\cdot c_{1}^{w}\cdot c_{2s}^{x}\cdot c_{2c}^{y}\cdot c_{3}^{z}$$

(13) $$f_{{cu}_{a}}=f_{{cu}_{c}}\cdot a_{1}^{w}\cdot a_{2s}^{x}\cdot a_{2c}^{y}\cdot a_{3}^{z}$$

where $f_{{cu}_{c}}$ and $f_{{cu}_{a}}$ are the theoretical values of I-RAPC compressive strength with concrete and acrylic pipe, respectively, MPa; *P* is the strength of reference group, MPa; *c*_1_, *a*_1_ are the strength reduction coefficients of concrete and acrylic middle pipes, respectively; *c*_2_, *a*_2_ are the strength reduction coefficients of the middle pipes in the concrete and acrylic laterals, respectively; *c*_2*s*_, *a*_2*s*_ are the located in the middle (top) part of the edge, respectively; *c*_2*c*_, *a*_2*c*_ are the located in the middle (side) part, respectively; *c*_3_, *a*_3_ are the strength reduction coefficients of corner pipes made of concrete and acrylic, respectively; *w*, *x*, *y*, *z* are the number of the different types of pipes, respectively.

**Figure 12:**
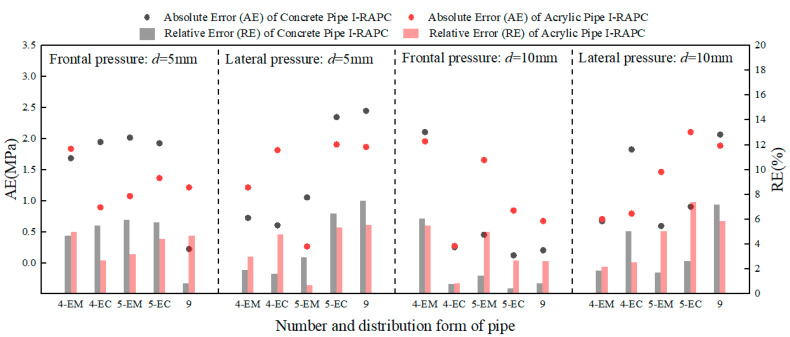
The error diagram between the calculated values and actual values of I-RAPC compressive strength.

The error between the calculated and actual values of I-RAPC compressive strength is illustrated in Figure 12. The absolute error range of I-RAPC compressive strength was 0.1~2.5 MPa, and the relative error range was 0.43~7.52%, as illustrated in the figure. As a result, the theoretical I-RAPC compressive strength calculation model proposed in this paper can accurately quantify and predict the reduction effect of pipe number, pipe diameter, and pipe distribution on I-RAPC compressive strength.

#### 3.2.3. Permeability Coefficient

**Figure 13:**
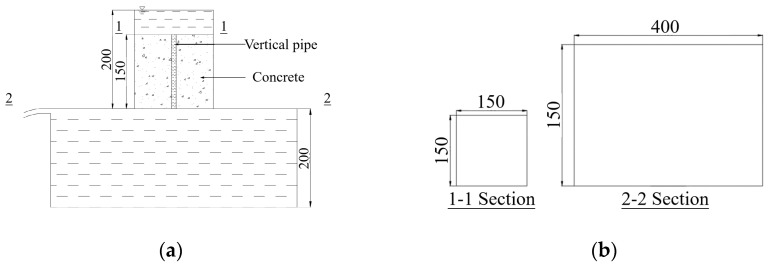
Calculation diagram of water permeability coefficient (unit: mm): (**a**) the water permeability device; (**b**) the size of Section 1–1, 2–2.

The permeability coefficient of I-RAPC was mainly determined by the number, diameter, and material (coefficient of roughness) of pipes. With the increase in the number and diameter of pipes, the coefficient of roughness decreased, and the permeability coefficient increased. Due to the unique characteristics of the upper- and lower-connecting pipes, the theoretical value of the I-RAPC permeability coefficient could be obtained by combining the free outflow method of simple short-pipe constant flow in hydraulics with Darcy’s law. The calculation process is seen in Figure 13.

In the calculation, the viscous dissipation of the water body was ignored, and only the influence of external boundary conditions was considered. That is, in the experiment, there are water head losses along the way and local head loss, so the flow *Q* of the free outlet pipe can be calculated using the following Equations (14) and (15):

(14) $$Q=\mu_{c}A\sqrt{2gH}$$

(15) $$\mu_{c}=\frac{1}{\sqrt{1+\lambda \cdot L/d+\sum \zeta }}$$

where Q is the flow, m^3^/s; $\mu_{c}$ is the flow coefficient of pipe; A is the cross-sectional area of the pipe, m^2^; g is the gravity acceleration, m/s^2^; H is the head difference, m; λ is the head loss coefficient along the course; L is the length of the pipe; d is the diameter of the pipe; ζ is the localized head loss coefficient.

In the equation above, the determination of and values were the key, which could be obtained from the hydraulic equation. The calculation and values are shown in Table 8 and Table 9.

.

According to Darcy’s law, the theoretical value of the I-RAPC permeability coefficient can be calculated as follows:

(16) $$k=\mu_{c}\sqrt{2g/H}\cdot LxA_{t}/A_{s}$$

where x is the number of pipes; $A_{t}$ is the water flow area of a single pipe, m^2^; $A_{s}$ is the top area of the I-RAPC, m^2^.

From Equation (16), the permeability coefficient was positively correlated with the flow coefficient and diameter of the pipe. When the roughness of the pipe was small, the flow coefficient of the pipe would increase. Therefore, embedding acrylic pipes would moderately increase the permeability coefficient of I-RAPC. At the same time, the influence of the pipe diameter on the permeability coefficient was more obvious. When the pipe diameter increased from 5 mm to 10 mm, the permeability coefficient could increase by more than four times. Comparing the theoretical value calculated with the actual value, the results are shown in Table 10. The absolute error range between the permeability coefficient of different pipe materials and diameters and the absolute error was 0.008~0.036 mm/s, and the relative error range was 0.265~4.420%. It could be seen that the theoretical calculation formula of the I-RAPC permeability coefficient deduced in this paper had high accuracy. In addition, it had been shown that the permeability coefficients of pervious concrete could reach 1.29 mm/s and 2.78 mm/s, respectively, when the compaction strength was 1.5 MPa [19] or the PPTF addition was 11 kg/m^3^ [43]. Compared to these studies, I-RAPC had a relatively high permeability coefficient when the pipe used was acrylic. This might be due to the fact that the concrete compaction process or the presence of fibers compressed some of the pore space, making the permeability coefficient of permeable concrete lower. In contrast, I-RAPC did not suffer from such problems.

## 4. Conclusions

The frontal- and lateral-compressive strength and permeability of innovative recycled aggregate pervious concrete (I-RAPC) were investigated in this paper under various pipe parameters, and a theoretical calculation model was constructed by response surface methodology (RSM). It was able to provide the foundation for its future development and application. The following are the primary conclusions:

(1) Based on the RSM, an optimization model of the I-RAPC was established, with the frontal- and lateral-compressive strength and the permeability coefficient as response values and the number of pipes, diameter, and material as response factors. After analysis and post hoc testing, it was determined that the model developed had a high degree of accuracy. The model regression equations were able to simulate more than 90% of the variation in response values, and could be used to predict the test results;

(2) All I-RAPC test groups in this article have compressive strength between 20 MPa and 45 MPa. The more pipes there are, the larger the diameter of the pipe, and the lower the compressive strength. The strength performance of a single pipe in the middle position is better than that in the corner position but the variation pattern of the permeability coefficient is opposite to that of compressive strength. When the number of pipes was nine and the diameter of the pipe was 10 mm, the permeability coefficient reached 27 mm/s;

(3) A new compressive strength calculation model of recycled aggregate pervious concrete based on the strength reduction coefficient method was proposed. The model could accurately determine the reduction effect of pipe number, pipe diameter, and pipe distribution on I-RAPC compressive strength. The maximum relative error between the calculated value and the actual value was only 7.52%;

(4) The calculation formula of the single-pipe permeability coefficient of the innovative recycled aggregate pervious concrete was deduced by the employment of the hydraulic method and Darcy’s law. The maximum relative error between the calculated value and the actual value was only 4.42%.

## Figures and Tables

**Table 1:** Index of RCA.

Aggregate Type	Particle Size (mm)	Apparent Density (kg·m^−3^)	Bulk Density (kg·m^−3^)	Sediment Percentage (%)	Water Content (%)	15 min Water Absorption (%)	Maximum Water Absorption Rate (%)	Crushing Index (%)	Numerical Tube Pressure (MPa)
RCA	4.75~9.5	2595	1245	0.25	2.80	4.70	5.42	15.54	1.7

**Table 2:** Mix design of I-RAPC.

Water Cement Ratio	Material Consumption Per Unit Volume/(kg·m^−3^)			
	Cement	RCA (4.75~9.5 mm)	Fine Aggregate	Water
0.49	418	1164	613	205

**Table 3:** Design of I-RAPC response surface test.

Experimental Levels			*x*: Number			*d*: Diameter			*E*: Elastic Modulus			*n*: Coefficient of Roughness		
						(mm)			(GPa)					
Low			1			5.0			1.00 (B)			0.0075 (A)		
Medium			5			7.5			2.33 (A)			0.0100 (C)		
High			9			10			3.00 (C)			0.0150 (B)		
Experimental data points		1	2	3	4	5	6	7	8	9	10	11	12	13~17
	*x*	1	9	1	9	1	9	1	9	5	5	5	5	5
*f_cu_*	*d*	5	5	10	10	7.5	7.5	7.5	7.5	5	10	5	10	7.5
	*E*	2.33	2.33	2.33	2.33	1	1	3	3	1	1	3	3	2.33
	*x*	1	9	1	9	1	9	1	9	5	5	5	5	5
*k*	*d*	5	5	10	10	7.5	7.5	7.5	7.5	5	10	5	10	7.5
	*n*	0.01	0.01	0.01	0.01	0.0075	0.0075	0.015	0.015	0.0075	0.0075	0.015	0.015	0.01

Notes: A: acrylic pipe; B: bamboo pipe; C: concrete pipe; *f_cu_*: compressive strength; *k*: permeability coefficient; *E*: the elastic modulus of pipes; *n*: the coefficient of roughness.

**Table 4:** The plan of I-RAPC compressive strength theoretical model construction.

Material	Diameter	Number	Distribution Form
Concrete (C) Acrylic (A)	5 mm 10 mm	1	C/EM/EC
		4, 5	EM/EC
		9	C, EM, EC

**Table 5:** Results of response model variance analysis.

Category		Sum of Squares	Degrees of Freedom	Mean Square Error	*F*	*p* Value *Pr > F*	
Frontal compressive strength	Model	259.2	7	37.0	25.7	<0.0001	Significant
	Lack of fit	10.0	5	2.0	2.7	0.1815	Insignificant
Lateral compressive strength	Model	162.3	7	23.2	28.8	<0.0001	Significant
	Lack of fit	4.7	5	0.9	1.5	0.3698	Insignificant
Permeable coefficient	Model	701.4	10	70.1	917.0	<0.0001	Significant
	Lack of fit	0.3	2	0.1	3.4	0.1385	Insignificant

**Table 6:** Regression equation error statistical analysis results.

Statistics Project	Frontal/Lateral Compressive Strength/Permeability Coefficient	Statistics Project	Frontal/Lateral Compressive Strength/Permeability Coefficient
*Std. Dev.*	1.20/0.90/0.28	*R-Squared*	0.9524/0.9573/0.9993
*Mean*	31.46/35.24/8.47	*Adj R-Squared*	0.9153/0.9240/0.9983
*C.V. %*	3.79/2.55/3.27	*Pred R-Squared*	0.7765/0.7445/0.9575
*PRESS*	60.82/43.32/29.86	*Adeq Precision*	16.57/22.923/113.46

**Table 7:** Reduction coefficient of I-RAPC compressive strength.

Coefficient Category	Pipe Material	Diameter/mm	Pipe Position			
			Center (C) *c*_1_	Edge Middle (EM) *c*_2_		Edge Corner (EC) *c*_3_
				Edge Middle Top (EM-T) *c*_2_*_T_*	Edge Middle Side (EM-S) *c*_2_*_s_*	
Reduction coefficient of frontal compressive strength	Concrete	5	0.9444	0.9485		0.9453
		10	0.9163	0.9453		0.9320
	Acrylic	5	0.9158	0.9558		0.9375
		10	0.9313	0.9521		0.9376
Reduction coefficient of lateral compressive strength	Concrete	5	0.9545	0.9660	0.9568	0.9628
		10	0.9478	0.9589	0.9553	0.9568
	Acrylic	5	0.9434	1.0245	0.9476	0.9663
		10	0.9447	1.0074	0.9407	0.9664

Notes: According to the compressive characteristics of I-RAPC, during frontal compressive strength test, the distribution forms of single pipes were C, EC, and EM (only one form); and during the lateral compressive strength, the distribution forms of single pipes were C, EC, and EM, respectively, and EM includes EM-T and EM-S.

**Table 8:** Calculation table of frictional head loss coefficient λ.

Pipe Material	Coefficient of Roughness	Diameter/mm	Hydraulic Radius/m	Chezy’s Coefficient C	Head Loss Coefficient along the Way λ
Concrete	0.01	5	0.00125	32.82	0.07285
		10	0.0025	36.84	0.05782
Acrylic	0.0075	5	0.00125	43.76	0.04098
		10	0.0025	49.12	0.03253

**Table 9:** Calculation table of local head loss coefficient ζ.

Section Number	Section Form	Pipe Diameter/mm	Section Area before Change/mm^2^	Section Area after Change/mm^2^	Local Head Loss Coefficient ζ
1–1	Sudden shrinkage	5	22,500	19.625	0.4996
		10	22,500	78.500	0.4983
2–2	Sudden expansion	5	19.625	120,000	0.9997
		10	78.500	120,000	0.9987

**Table 10:** Theoretical and actual values of I-RAPC water permeability coefficient of a single pipe.

Pipe Material	Pipe Diameter (mm)	Flow Coefficient *μ_c_*	Flow *Q* (cm^3^/s)	Permeability Coefficient *k* (mm/s)		Error	
				Calculated Value	Actual Value	Absolute Error (mm/s)	Relative Error (%)
Concrete	5	0.5276	20.773	0.692	0.724	0.032	4.420
	10	0.5452	85.832	2.861	2.897	0.036	1.243
Acrylic	5	0.5668	22.312	0.744	0.754	0.010	1.326
	10	0.5763	90.726	3.024	3.016	0.008	0.265

## Data Availability

The original contributions presented in the study are included in the article, further inquiries can be directed to the corresponding author.
